# Epigenetic Regulation of miRNA-211 by MMP-9 Governs Glioma Cell Apoptosis, Chemosensitivity and Radiosensitivity

**DOI:** 10.18632/oncotarget.683

**Published:** 2012-11-16

**Authors:** Swapna Asuthkar, Kiran Kumar Velpula, Chandramu Chetty, Bharathi Gorantla, Jasti S. Rao

**Affiliations:** ^1^ Departments of Cancer Biology and Pharmacology, University of Illinois College of Medicine at Peoria, Peoria, IL, U.S.A; ^2^ Departments of Neurosurgery, University of Illinois College of Medicine at Peoria, Peoria, IL, U.S.A

**Keywords:** Matrix metalloproteinase-9 (MMP-9), microRNA-211 (miR-211), Cancer Stem Cells (CSC), Ionizing radiation (IR), Temozolomide (Tem)

## Abstract

Glioblastoma multiforme (GBM) is the most aggressive brain cancer, and to date, no curative treatment has been developed. In this study, we report that miR-211, a microRNA predicted to target MMP-9, is suppressed in grade IV GBM specimens. Furthermore, we found that miR-211 suppression in GBM involves aberrant methylation-mediated epigenetic silencing of the miR-211 promoter. Indeed, we observed a highly significant inverse correlation between miR-211 expression and MMP-9 protein levels, which is indicative of post-transcriptional control of gene expression. Additionally, shRNA specific for MMP-9 (pM) promoted miR-211 expression via demethylation of miR-211 promoter-associated CpG islands (-140 to +56). In independent experiments, we confirmed that miR-211 overexpression and pM treatments led to the activation of the intrinsic mitochondrial/Caspase-9/3-mediated apoptotic pathway in both glioma cells and cancer stem cells (CSC). We also investigated whether miR-211 is involved in the regulation of MMP-9 and thus plays a functional role in GBM. We found an acute inhibitory effect of miR-211 on glioma cell invasion and migration via suppression of MMP-9. Given the insensitivity of some GBMs to radiation and chemotherapy (temozolomide) along with the hypothesis that glioma CSC cause resistance to therapy, our study indicates that miR-211 or pM in combination with ionizing radiation (IR) and temozolomide significantly induces apoptosis and DNA fragmentation. Of note, miR-211- and pM-treated CSC demonstrated increased drug retention capacity, as observed by MDR1/P-gp mediated-Rhodamine 123 drug efflux activity assay. These results suggest that either rescuing miR-211 expression or downregulation of MMP-9 may have a new therapeutic application for GBM patients in the future.

## INTRODUCTION

Transcriptional and post-transcriptional regulators of gene expression, including microRNAs (miRNAs), are being investigated as a relatively new and important class of oncogenes and tumor suppressors [1-5]. The miRNAs comprise a class of evolutionarily conserved small RNAs (22 nts long) that affect gene expression at the post-transcriptional level by blocking translation or degrading target messenger RNAs (mRNAs) [6, 7]. Although discovered only recently, miRNAs have been found to influence cancer development in many ways, including the regulation of cell proliferation, cell transformation, and programmed cell death [2, 8]. Several studies have reported that miRNA genes are often found in genomic regions linked to cancer [9, 10], and miRNA expression profiles are correlated with the developmental lineage and the differentiation state of tumors [11].

In this study, we focused on miRNA-211 (miR-211), which plays a functional role in the regulation of apoptosis and tumor progression. The expression of miR-211 is greatly decreased in melanoma cells and melanoblasts compared to melanocytes, and recent studies showed that overexpression of miR-211 causes suppression of tumor invasion in melanoma [12, 13]. Using experimental and bioinformatic approaches, we selected miR-211, a commonly downregulated miRNA, and analyzed its relationship to the expression of glioblastoma antigen (MMP-9), which is also a potential target of miR-211. Matrix metalloproteinase-9 (MMP-9) acts as an important oncogene that enhances the invasiveness of cancer cells [14], and prior research demonstrates that a high level of MMP-9 confers a poor prognosis in a variety of cancers [15, 16]. Earlier studies also showed that the siRNA-mediated knockdown of MMP-9 expression in glioma cells induces apoptosis and inhibits tumor growth [17, 18]. Apart from its key role in tumor invasion, the elevated expression of MMP-9 in different cancers is also implicated in increased tumor progression, including metastasis and shorter survival times [16, 19-21]. Recently, the MMP-9-specific miRNA expression profile was established via miRNA microarrays as a potential target of anti-invasion therapy in glioblastoma [22]. Two miRNAs, miR-885-5p and miR-491-5p, were determined to reduce the level of MMP-9 and inhibit cellular invasion in U251 and U87 glioma cells. In the present study, we investigated the functional role of miR-211 in inducing glioma cell apoptosis via suppression of MMP-9. Moreover, we observed that the shRNA-mediated downregulation of MMP-9 (pM) resulted in significant upregulation of miR-211 in GBM cells. Recently, case report studies on childhood glioblastoma suggested that MMP-9 upregulation could be associated with tumor resistance to temozolomide at relapse [23]. In the present study, we demonstrated that either the downregulation of MMP-9 (pM) or overexpression of miR-211 increases chemo- and radio-sensitivity of glioma CSC, suggesting that rescuing miR-211 expression may have a new therapeutic application in the treatment of GBM patients in the future. We also provide preliminary evidence of the epigenetic control of miR-211 by MMP-9 overexpression in GBM but the mechanisms underlying this regulation needs further investigation.

## RESULTS

### The miR-211 levels inversely correlate with MMP-9 protein in GBM cells and MMP-9-specific methylation of CpG island region (-140 to +56) of miR-211 promoter

**Figure 1:**
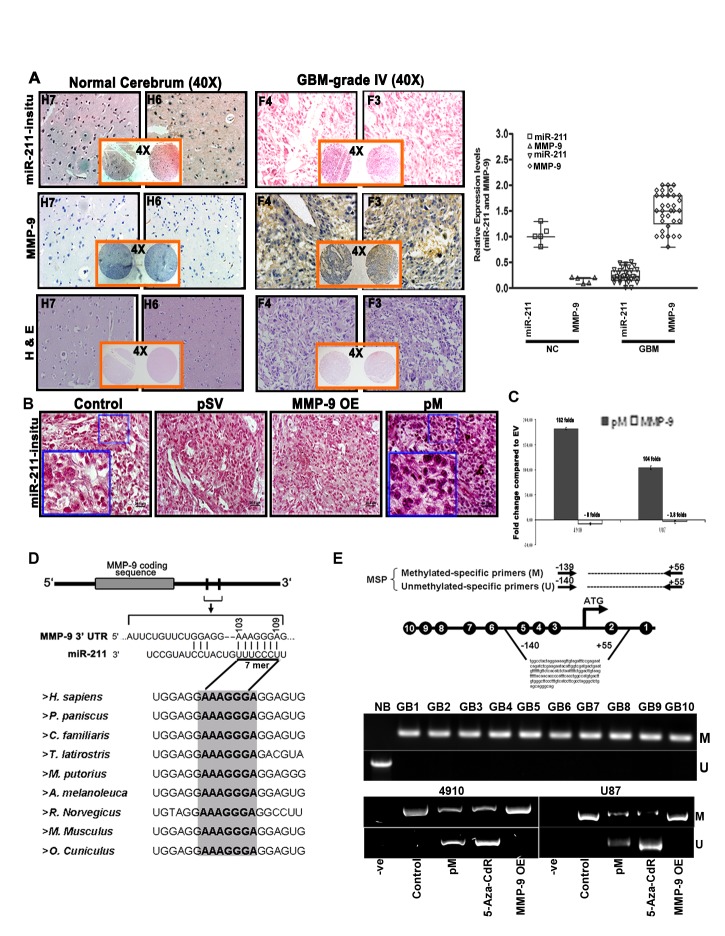
miR-211 negatively regulates MMP-9 in GBM and methylation of the CpG island (-140 to +56) promoter region of miR-211 is associated with MMP-9 (A) Hematoxylin and eosin (H&E) staining of brain sections displaying the neoplastic growth of tumors were further analyzed through *in situ* and DAB staining to detect the basal expression levels of miR-211 and MMP-9 respectively in normal brain (H6 & H7) and GBM grade IV (F3 & F4) specimens. The relative expression of miR-211 and MMP-9 protein in GBM tissue microarray (containing 33 cases of brain glioblastoma and 5 normal brain tissues, duplicate cores per case) was quantified and is represented graphically (*p<0.001*). (B) *In situ* hybridization studies showing the expression of miR-211 in intracranial tumors induced by control, pSV-, MMP-9 OE-, and pM-treated 4910 cells. (C) Relative expression levels of miR-211 using stem and loop primers by miR-quantitative RT-PCR analysis in control, pM-, and MMP-9 OE-treated 4910 and U87 cells (mean + SD, n=3; *p<0.001*). (D) Sequence alignment of miR-211 and the predicted sequence pairing with a region of MMP-9 mRNA 3'UTR that may interact with miR-211. Alignment of nucleotide sequences of MMP-9 3′-UTR corresponding to the targets for miR-211 across nine different species. A high level of conservation suggests a functional role for these sequences. (E) Schematic map of the miR-211 promoter-associated CpG island region indicating the location of PCR primers used for MSP analysis. Each of the CpG dinucleotide residues with respect to the transcriptional start site ATG is shown as numbers in circles from 1 to 10, which can serve as potential DNA methylation sites in miR-211 promoter. Four potential CpG sites (5/-111 to 2/+33, genomic positions 49731, 49844, 49860 and 49874; Accession No. AEKP01201896) were investigated by MSP as mentioned in Materials and Methods. Bisulfite-modified DNA derived from human glioblastoma grade IV specimens (GB1-GB10) was amplified using MSP primers and was compared with normal human brain specimen (NB). MSP assay was also performed on DNA isolated from control, pM, 5-Aza-CdR and MMP-9 OE-treated 4910 and U87 cells. The bisulfite-modified DNA was amplified with miR-211 promoter primers specific for methylated (top) and unmethylated DNA (bottom). Methylation-specific primers generated the PCR products labeled with “M.” Those labeled with “U” were generated by primers specific for unmethylated DNA.

To determine the basal expression levels of miR-211 in glioblastoma tissue specimens, we first performed *in situ* hybridization analysis to examine the expression levels of miR-211 in a GBM tissue microarray containing 33 specimens of glioblastoma and 5 specimens of normal brain tissue, duplicate cores per case. Fig. 1A shows that the GBM tissue specimens (F3 and F4) have low endogenous miR-211 levels when compared to the normal cerebrum tissues (H6 and H7). Further, the immunohistochemistry analysis performed to examine the MMP-9 protein levels in the GBM specimens demonstrated an inverse correlation between miR-211 expression and MMP-9 protein levels. The GBM tissues with low miR-211 levels showed significantly higher expression of MMP-9 protein; conversely, the normal cerebrum (NC) tissues with high levels of miR-211 showed lower expression of MMP-9 protein (Fig. 1A). Overall, the analysis showed that the miR-211 level was inversely correlated with MMP-9 level in a total of 25 GBM specimens (*p<0.001*). Further, the *in situ* hybridization analysis of intracranial tumors obtained from 4910 xenograft cells in nude mice also revealed low endogenous levels of miR-211 in GBM control cells (Fig. 1B). Interestingly, the intracranial brain tumors treated with shRNA specific for MMP-9 (pM) showed noticeably increased miR-211 levels. However, the expression levels of miR-211 remained low in the MMP-9 overexpression construct (MMP-9 OE) treated intracranial mice brain tumors (Fig. 1B). For further *in vitro* investigation into the inverse correlation between MMP-9 protein and miR-211, we examined the relative expression levels of miR-211 using miR-quantitative stem-loop PCR analysis in empty vector (EV), pM and MMP-9 OE-treated 4910 and U87 cells. We observed that the downregulation of MMP-9 resulted in a significant increase in miR-211 levels whereas the overexpression of MMP-9 showed little decrease or no obvious differential expression of miR-211 (Fig. 1C). Moreover, from the miRanda [24] and TargetScan [25] software for microRNA target prediction, we found that miR-211 targets 103-109 nts of 3'-UTR of MMP-9, which is highly conserved across nine species (Fig. 1D). These data suggest that miR-211 may target oncoproteins, such as MMP-9, and act as a tumor suppressor in GBM cells.

To further investigate the downregulation of miR-211 in GBM cells, we considered an alternative mechanism of miRNA downregulation by epigenetic transcriptional silencing [26]. We analyzed the DNA methylation status at known or predicted transcriptional start sites (TSSs) of the miRNA-211 promoter in ten human grade IV GBM tissues (GB1-GB10) (from surgical biopsy specimens) by methylation-specific PCR (MSP) analysis. The genomic DNA from the tumor tissues was subjected to bisulfite treatment, which converts unmethylated cytosine to uracil while leaving 5-methylcytosine unchanged. Two methylation-specific primer sets were designed to amplify either methylated (M) or unmethylated (U) bisulfite-modified sequence in the miR-211 promoter (Fig. 1E). Of note, all the GBM specimens showed aberrant miR-211 promoter hypermethylation when compared to normal brain (NB) tissue, which showed no evidence of promoter hypermethylation. These results suggest the presence of dense methylation of the miR-211 promoter region (-140 to +56) in GBM cells. Further, we also investigated whether the negative correlation between MMP-9 and miR-211 expression was associated with altered DNA methylation of miR-211 promoter. We analyzed the DNA methylation status of miR-211 promoter region (-140 to +56) in control, pM, 5-Aza-CdR and MMP-9 OE-treated 4910 and U87 cells and found that the control and MMP-9 OE cells showed prominent PCR products with the methylated primer but no products were detected with the unmethylated primer. Interestingly, the active demethylating agent, 5-Aza-CdR-treated cells and the cells knocked down for MMP-9 (pM) showed methylation-specific PCR (MSP) products in the unmethylated lane, indicating demethylation of miR-211 promoter CpG island (Fig. 1E). The demethylation of miR-211 promoter with pM treatment suggests transcriptional activation of miR-211, which is consistent with our *in situ* hybridization and miR-quantitative PCR analysis (Figs. 1B and 1C).

### Both miR-211 overexpression (miR-211) as well as MMP-9 downregulation (pM) affect cell viability and induce apoptosis in GBM cells

**Figure 2:**
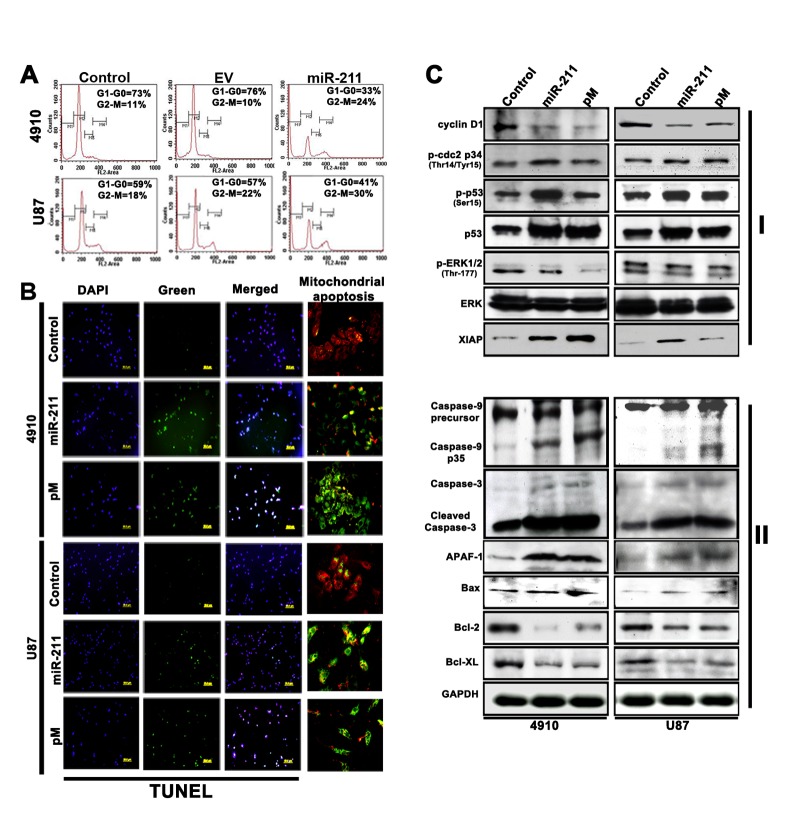
miR-211 induces apoptosis in human xenograft cell lines (A) Fluorescence-activated cell sorting (FACS) analysis of cell cycle progression in 4910 and U87 xenograft cells 48 h after miR-211 transfection. The *y* axis denotes cell count and the *x* axis represents DNA content. The percentages of cells in the G1 (M2), S (M3) and G2/M (M4) phases of the cell cycle were calculated using CellQuest Pro software. EV represents the empty vector control. (B) Whole cell lysates were prepared from control, miR-211 and pM-treated cells and subjected to western blot analysis to determine the expression of various cell cycle (Panel I), pro-apoptotic and anti-apoptotic (Pane II) proteins. GAPDH was used as a loading control. (C) The 4910 and U87 xenograft cells were seeded in 8-well chamber slides and transfected with miR-211 or pM and incubated further for 48 h. The cells were fixed in 4% paraformaldehyde and subjected to TUNEL assay as described in Materials and Methods. Green fluorescence represents apoptotic cells and blue (DAPI) fluorescence represents the nucleus. Mitochondrial apoptosis in control, miR-211 and pM-treated 4910 and U87 cells was examined by MitoLight® Mitochondrial Apoptosis Detection Kit as described in Materials and Methods. Only the merged figures are represented for mitochondrial apoptosis. In healthy cells, the dye accumulates and aggregates in the mitochondria, giving off a bright red fluorescence. In apoptotic cells with altered mitochondrial membrane potential, the dye in its monomeric form stays in the cytoplasm, fluorescing green, providing a ready discrimination between apoptotic and nonapoptotic cells.

To determine whether the overexpression of miR-211 in GBM cells, with lower endogenous miR-211 levels, can affect MMP-9 protein expression and cell viability, the 4910 and U87 cells were transiently transfected with a miR-211-overexpressing plasmid. The cell cycle progression was analyzed by FACS (Fig. 2A), which demonstrated a significant decrease in G0/G1 phase of 4910 and U87 cells with miR-211 overexpression when compared to control and empty vector (EV)-treated cells. This concomitant decrease in the percentage of cells in G_1_ phase was associated with significant cell cycle arrest in G_2_/M phase, with a smaller reduction in the S phase population (Fig. 2A). Earlier studies in our lab have demonstrated the role of shRNA specific for MMP-9 (pM) in the induction of G2-M arrest and apoptosis in cancer cells [18, 27]. However, G2-M arrest precedes apoptosis [28] and after the G_2_-M arrest tumor cells are known to exhibit morphologic changes consistent with apoptosis [29]. To further evaluate the role of miR-211 and pM in the induction of apoptosis in glioma cells, GBM cells were transfected with miR-211 and pM plasmids. We observed that the miR-211 and pM treatments decreased the number of cells in the culture dishes. To confirm whether the decrease in the number of viable cells following miR-211 and pM treatments was due to the induction of apoptosis, we stained the cells with TUNEL. We found that the numbers of cells stained with TUNEL were higher following the miR-211 and pM treatments (Fig. 2B). Next, immunoblotting was performed to detect the altered expression of cell cycle regulatory (Fig. 2C, Panel I) and apoptosis-related molecules (Fig. 2C, Panel II) in miR-211- and pM-treated cells when compared to control 4910 and U87 cells. The immunoblot analysis showed that the expression levels of cell proliferation and survival-associated molecules, such as cyclin D1, ERK and pERK were decreased whereas the levels of cell cycle inhibitors, such as p-cdc2, p53, p-p53 and XIAP were increased with miR-211 and pM treatments (Fig. 2C Panel I). The pivotal role of MMP-9 in enhancing glioma tumor migration, invasion and angiogenesis has already been well studied [30]. Furthermore, the role of MMP-9 inhibition (pM) in inducing Caspase-9-mediated apoptosis in SNB19, a GBM cell line, has been shown earlier [31]. The immunoblot results obtained from the suppression of MMP-9 and overexpression of miR-211 in 4910 and U87 cells were comparable and elicited similar anti-proliferative and apoptotic signaling mechanisms. The results demonstrated a significant increase in the fragmentation and proteolytic activity of Caspase-9 with miR-211 and pM treatments when compared to the control cells. In particular, the cleaved 35 kDa fragment of Caspase-9 increased, which suggests the activation of Caspase-9 after the treatments. The activated Caspase-9 then triggers pro-apoptotic pathways through the proteolytic cleavage and activation of effector caspases, such as Caspase-3 (Fig. 2C Panel II). Caspases have been shown to contribute to mitochondrial disruption during apoptosis [32] and further we tested whether Caspase-9 activation was involved in this process using the MitoLight™ mitochondrial apoptosis detection kit. We observed that both the overexpression of miR-211 as well as suppression of MMP-9 caused mitochondrial apoptosis in GBM cells (Fig. 2B). The mitochondrial disruption along with the activated effector caspases enhances the apoptosis signal through enzyme activation and degradation of hundreds of intracellular protein targets [33]. Apart from activation of Caspase-9, we also observed a significant increase in the apoptosome-associated molecule, Apaf-1, which suggests the activation of the mitochondrial/Caspase-9/3-mediated apoptotic pathway. Oligomerization of the pro-apoptotic multi-domain Bcl-2 family members, such as Bax, in the outer mitochondrial membrane seems to be obligatory to initiate mitochondrial dysfunction in most apoptotic stimuli. In contrast, anti-apoptotic Bcl-2 family members, such as Bcl-2 and Bcl-xL, appear to stabilize mitochondria by inhibiting the formation of such oligomers. Importantly, the ratio of pro- and anti-apoptotic protein expression, such as Bax/Bcl-2 determines a cell's susceptibility to undergo apoptosis [34, 35]. In the present study, the constitutive expression of anti-apoptotic proteins Bcl-2 and Bcl-xL decreased after miR-211 and pM treatments, whereas the expression of the pro-apoptotic protein Bax increased, thereby demonstrating a shift in Bax/Bcl-2 ratio in favor of apoptosis in treated cells when compared to the control cells. These observations show that both the miR-211 and pM treatments induce apoptosis, which is primarily p53-dependent and mediated through the activation of the Caspase-9/3 pathway (Fig. 2C Panel II).

### miR-211 suppresses MMP-9 expression levels and sensitizes glioma stem cells to ionizing radiation (IR)

**Figure 3:**
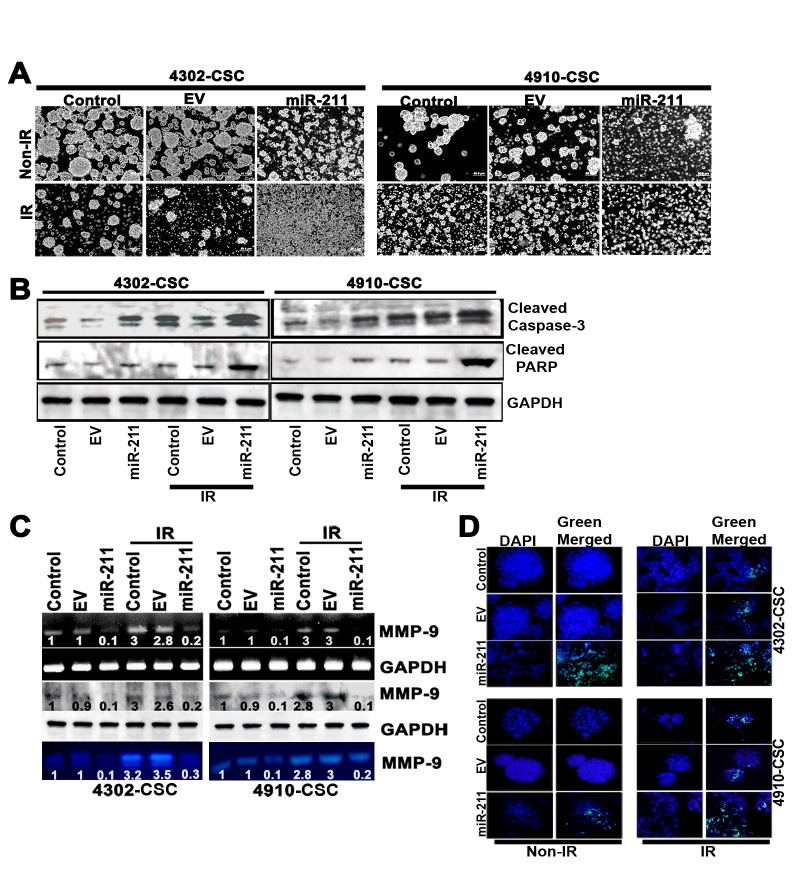
miR-211 inhibits radiation-induced MMP-9 and CSC-like property in glioma cells (A) Effect of miR-211 overexpression on the ionizing radiation (IR)-treated glioma 4302 and 4910 CSC spheres. (B) Immunoblots using cell lysates (40 μg) from control, empty vector (EV), miR-211 alone and in combination with IR-treated 4302 and 4910 CSC were developed using antibodies specific for cleaved Caspase-3 and cleaved PARP. GAPDH was used as a loading control. (C) MMP-9 mRNA and protein levels were examined by RT-PCR and immunoblot analysis respectively. Conditioned medium, collected from the above treated glioma CSC was subjected to gelatin zymography to determine MMP-9 activity. (D) TUNEL nuclear staining in 4302 and 4910 CSC spheres. The control CSC with overexpression of miR-211 alone and in combination with IR treatment in 8-well chamber slides were subjected to TUNEL nuclear staining and viewed by fluorescence microscopy. Green fluorescence represents apoptotic cells and blue (DAPI) fluorescence represents the nucleus.

Since radiation and multi-drug resistance are the characteristic properties of cancer stem cells (CSC) and tumor initiating cells of various tumor origins, there is increasing interest in establishing a clear association between miRNA expression in tumors and chemo- and radiosensitivity, with regard to predicting or modulating sensitivity [36]. To examine the effect of miR-211 on the disintegration of glioma CSC spheres, we conducted sphere disintegration assay followed by 5 days of IR treatment. The results showed a remarkable increase of sphere disintegration with miR-211 treatment. However, the greatest effect of disintegration was observed in miR-211 treatment accompanied with ionizing radiation (IR) (Fig. 3A), suggesting that miR-211 is more effective in inhibiting the functions of cancer stem-like cells. To confirm whether the increased sphere disintegration following miR-211 treatment was due to apoptosis, we performed immunoblot analysis of 4302 and 4910 control and miR-211-treated CSC alone and in combination with IR treatment. We assessed proteolytic cleavage and activation of Caspase-3 and cleaved PARP. The overexpression of miR-211 showed significant increase in the levels of cleaved Caspase-3 and PARP, which was further enhanced in combination with IR treatment (Fig. 3B). Since miR-211 levels inversely correlate with MMP-9 protein levels, we examined the expression levels of MMP-9 in miR-211 and IR-treated glioma CSC using RT-PCR and immunoblot analyses. We observed that with the IR treatment, the total levels of MMP-9 increased *(p<0.01)* when compared to control CSC. Notably, this IR-induced MMP-9 protein was suppressed upon miR-211 overexpression. Furthermore, the MMP-9 activity, as examined by gelatin zymography, increased with IR treatment whereas, this IR-induced MMP-9 activity was retarded by 60% and 59% in miR-211 overexpressing 4302 and 4910 CSC respectively. These results suggest that the inhibitory effect of miR-211 on glioma cell proliferation, at least in part, is mediated via suppression of MMP-9 (Fig. 3C). We also confirmed the miR-211-induced apoptosis in CSC spheres of 4302 and 4910 cells using TUNEL assay (Fig. 3D).

### miR-211 retards migration and invasion and suppresses TGF-β/Smad-1 signaling in IR-treated glioma CSC

**Figure 4:**
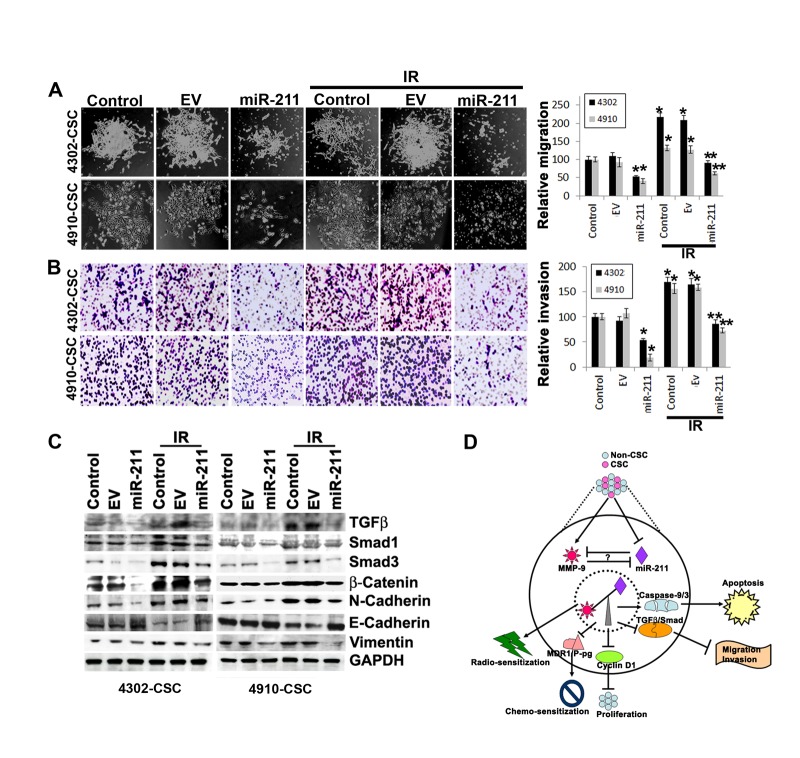
miR-211 suppresses the migratory and invasive potential of glioma CSC (A) 4302 and 4910 CSC spheres were seeded in 24-well plates. The CSC spheroids were either treated with empty vector (EV) or miR-211 alone or in combination with IR treatment. After 16 h, the media was replaced with fresh complete media. The spheroid migration distance was measured after 48 h and is represented graphically in terms of relative migration (*, *p<0.01*; **, *p<0.001*). (B) The treated 4302 and 4910 CSC were disintegrated into single cell suspension by trituration and an equal number (5×10^4^) of spheres were suspended in serum-free media and plated onto Matrigel-coated Transwell inserts as described in Materials and Methods. After a 24-hr incubation period, lower invaded spheres were stained with Hema-3. Images of invaded spheres were taken under a light microscope (Olympus IX-71). The invasive potential of treated spheres was quantified and the percentage of cells invading from 3 independent experiments is graphically represented as bar diagrams. Error bar represents mean + standard deviation (SD) (*, *p<0.01*; **, p<0.001). (C) Immunoblot analysis of cell lysates (40 μg) from control, EV and miR-211-treated 4302 and 4910 CSC to assess the expression of various EMT-associated proteins. GAPDH was used as a loading control. (D) Schematic representation shows that miR-211 mediates negative regulation of MMP-9 and plays a functional role in inhibition of glioma cell proliferation, migration and invasion. The miR-211, which is predicted to target MMP-9, is suppressed in GBM grade IV specimens. Furthermore, we observed that downregulation of MMP-9 using shRNA specific for MMP-9 (pM) promoted miR-211 expression via demethylation of miR-211 promoter-associated CpG island (-140 to +56). In light of these observations, we interpret a negative feedback loop between miR-211 and MMP-9 and suggest that either rescuing miR-211 expression or downregulation of MMP-9 may have a new therapeutic application for the treatment of GBM patients in the future.

The upregulation of MMP-9 is particularly important for the induction of cancer cell invasion and migration [37, 38], and suppression of MMP-9 in GBM cells significantly inhibits the invasion, migration and metastatic ability of these cells [17, 39]. Since the overexpression of miR-211 suppressed MMP-9 protein and showed inverse correlation, we investigated further the miR-211 role in glioma cell migration and invasion. We compared the migration and invasive ability of control 4302 and 4910 CSC to those cells treated with miR-211 alone and in combination with IR (Figs. 4A and 4B). The IR-treated glioma CSC showed increased spheroid migration and invaded through Matrigel more extensively when compared to the equal number (5×10^4^) of control CSC. However, miR-211 in combination with IR treatment significantly decreased the IR-induced migratory and invasive ability of glioma CSC (Figs. 4A and 4B). We further investigated the miR-211-mediated mechanisms that alter invasive and metastatic potential of 4302 and 4910 CSC. The initiation of metastasis via migration and invasion has many phenotypic similarities to epithelial mesenchymal transition (EMT) [40]. EMT is characterized by the loss of epithelial marker E-cadherin and the gain of mesenchymal markers, such as N-cadherin, Vimentin and β-catenin. The immunoblot analysis of cells treated with miR-211 alone and in combination with IR showed suppression of N-cadherin, Vimentin and β-catenin expression whereas showed increased expression of E-cadherin when compared to the control 4302 and 4910 CSC. In contrast, we observed that IR induced the expression of N-cadherin, Vimentin and β-catenin proteins in glioma CSC (Fig. 4C). These results suggest that the miR-211 suppresses EMT by inducing MET-like phenotype in glioma CSC. The western blot results also demonstrated reduced basal levels of Smad-1, Smad-3 and TGF-β proteins in miR-211 and IR-treated cells when compared to control glioma CSC (Fig. 4C).

### MiR-211; a modifier of chemotherapy in glioma CSC

**Figure 5:**
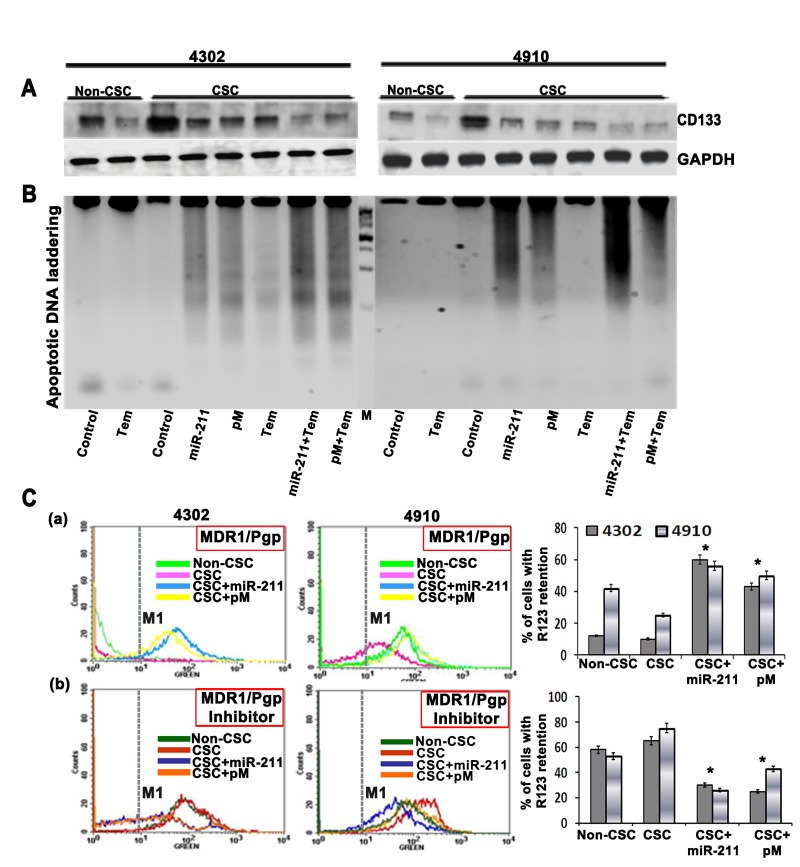
(A) 4302 and 4910 cancer stem cells (CSC) were treated with a chemotherapy compound, temozolomide (500 uM), or transfected with miR-211 and pM plasmids Immunoblot analysis was performed to determine CD133 levels in the treated CSC, and the results were compared with untreated CSC and normal cancer cells (non-CSC). GAPDH was used as a loading control. (B) Apoptotic DNA laddering profile of temozolomide, miR-211 and pM-treated glioma CSC and non-CSC. (D) Rhodamine 123 efflux assay to determine the role of miR-211 and pM in chemosensitivity. The assay measures P-gp-mediated efflux by the decrease in the intracellular fluorescence of rhodamine 123 (R123, a MDR1/P-pg substrate) using flow cytometry. Intracellular rhodamine 123 was measured at time 0 (load) and after 2 h of efflux in control, miR-211- and pM-treated glioma CSC (panel a). The efflux was measured by the number of cells in the M1 region of the plot. To confirm the specificity of the R123 efflux from the control and treated CSC, we employed a P-gp blocker, vinblastin. The R123 efflux assays were performed in the presence of this inhibitor (panel b). The percentage of cells with R123 retention was quantified both in the absence (panel a) and in the presence of P-gp blocker, vinblastine (panel b) from 3 independent experiments and is graphically represented as bar diagrams. Error bar represents mean + standard deviation (SD) (*, *p<0.01*).

The fraction of tumor cells expressing CD133 (Prominin-1), a marker for both neural stem cells and brain cancer stem cells, display strong capability on tumor's resistance to chemotherapy including temozolomide. These drug resistant CD133-positive cells also show higher expression of the anti-apoptosis and inhibitors of apoptosis protein families [41]. Studies have shown that CD133-expressing glioma cells survive ionizing radiation and chemotherapy in increased proportions relative to most tumor cells that lack CD133 [42-44]. In the present study, we observed increased CD133 expression in glioma CSC (*p<0.001*) when compared to glioma parental cells (non-CSC). This CSC-induced CD133 expression was significantly inhibited upon treatment of 4302 and 4910 CSC with miR-211 and pM alone and in combination with temozolomide (Fig. 5A). CSC are known to contribute to glioma chemoresistance through preferential activation of the DNA damage checkpoint response and an increase in DNA repair capacity [45]. Since miR-211 and pM treatments caused increased apoptosis via caspase-9/3 activation in glioma cells and CSC (Figures 2 and 3), we examined the apoptotic DNA laddering profile of miR-211- and pM-treated glioma CSC alone or after subjecting them to a sublethal dose of temozolomide. The miR-211- and pM-treated glioma CSC showed increased apoptotic DNA fragmentation when compared to the temozolomide-treated CSC, thus confirming their significant role in apoptotic induction (Fig. 5B). The combination treatments of miR-211 and pM with temozolomide showed low levels of CD133 and significantly enhanced apoptotic DNA fragmentation (Fig. 5A and 5B). The classic multi-drug resistance (MDR) mechanism involves alterations in the expression of highly evolutionarily conserved plasma membrane glycoproteins, P-glycoprotein (P-gp) and the multi-drug resistance protein (MRP) that actively transports drugs out of the cell or microorganism [46, 47]. The expression levels of P-gp and MRP have been shown to be increased after chemotherapy in GBM patients [48]. To further determine the role of miR-211 and pM in chemosensitivity, we performed the rhodamine 123 efflux assay. The assay measures P-gp-mediated efflux by the decrease in the intracellular fluorescence of rhodamine 123 (R123, a MDR1/P-pg substrate) using flow cytometry. Intracellular rhodamine 123 was measured at time 0 (load) and after 2 h of efflux in control, miR-211 and pM-treated glioma CSC (Fig. 5C panel a). The efflux was measured by the number of cells in the M1 region of the plot. We found enhanced R123 efflux from the glioma CSC, which indicates greater P-gp activity, when compared to the normal glioma cells (non-CSC). The miR-211 (CSC + miR-211) and pM (CSC + pM) treatments showed significant retention of R123 in CSC, suggesting lower P-gp activity. To confirm the specificity of the R123 efflux from the control and treated CSC, we employed a P-gp blocker, vinblastin. The R123 efflux assays were performed in the presence of this inhibitor, and the results are shown in Figure 5C panel b. The vinblastin at concentrations of 22 uM was able to significantly reduce the efflux of R123 from the glioma CSC, thus confirming that the decrease in efflux from miR-211- and pM-treated CSC was due to a decrease in P-gp expression and activity. These results suggest that targeting miR-211 and/or MMP-9 in cancer stem cells may overcome this radio- and chemoresistance and provide a therapeutic model for malignant GBM.

## DISCUSSION

Glioblastoma multiforme (GBM) is the most aggressive brain cancer, and no curative treatment is currently available. Despite improved surgery and chemo-radiotherapy approaches, the median survival time for patients with GBM remains less than one year [49]. The dismal prognosis of GBM is mainly due to the poor response of patients to any therapeutic modality. Thus, it is very important to find new biomarkers that can predict clinical outcomes and make good potential drug targets. MicroRNAs are small, non-coding RNAs that function as post-transcriptional regulators of gene expression and play a key role in pathogenesis of GBM [50]. Although discovered only recently in the early 1990s, the expression of miRNAs has been demonstrated to correlate with clinical outcomes in GBM. Despite the fact that there is only limited data available so far, these initial results are very promising and indicate that miRNAs might open a new perspective for diagnostics and treatment of different cancers, including GBM. In the present study, we demonstrated that miR-211 expression is suppressed in grade IV GBM specimens and the expression of miR-211 was inversely related to MMP-9 protein levels. We found that the overexpression of miR-211 in glioma cell lines retards MMP-9 activity, induces apoptosis and DNA damage response (DDR). In the last few years, the important role of the DDR pathway in tumor formation and modulation of therapeutic response has been appreciated. One of the primary reasons for treatment failure in GBM is local invasion, and recently, there has been an increase in information regarding specific molecules that determine the aggressiveness and invasion potential of high-grade astrocytic tumors. In particular, expression levels of matrix metalloproteases (MMPs) in high-grade gliomas appear to correlate with tissue invasiveness and are prognostically significant. The overexpression of MMP-9 in the invasive front of different malignant tumors is linked to poor clinical outcome. Previous studies have demonstrated that downregulation of MMP-9 not only reduced the invasive nature but also subsequently led to ERK and AKT-mediated apoptosis in meningioma and medulloblastoma cells [51, 52]. The siRNA-mediated knockdown of MMP-9 expression has also been shown to induce Caspase-9-mediated apoptosis in glioma cells [31]. Molecular analysis of the signaling cascades that lead to apoptosis is essential for delineating cell death events and for the development of novel cancer therapeutics. The results of the present study showed that both the overexpression of miR-211 (miR-211) and suppression of MMP-9 (pM) induced activation of intrinsic mitochondrial/Caspase-9/3-mediated apoptotic pathway. Furthermore, the overexpression of miR-211 significantly suppressed MMP-9 activity in glioma cells, as demonstrated by gelatin zymography. Thus, we assume that the anti-proliferative, anti-invasive and apoptotic effect of miR-211 on glioma cells is associated with the downregulation of MMP-9 activity.

More than 90% of cancer patient mortality is attributed to metastasis [53] and both *in vitro* and *in vivo* observations indicate that apoptosis is an important process regulating metastasis [54]. When we examined the metastatic ability of glioma cells after miR-211 and pM treatments, we found downregulation of TGF-β/Smad signaling. TGFβ can promote cancer metastasis through its effects on the tumor microenvironment by enhancing invasive properties and inhibiting immune cell function. The downstream transducers of the TGF-β pathway, the Smads, are also altered in cancer tissues. Additionally, TGF-β can regulate the expression, secretion, and activity of matrix metalloproteases MMP-2 and MMP-9. The Smad-dependent, TGF-β pathway plays a key role in the induction of EMT, and vvarious studies have explored the roles of TGF-β-activated Smads in EMT [55, 56]. Further, TGF-β-induced EMT often coincides with the loss of E-cadherin expression [57, 58]. In the present study, miR-211 and pM treatments decreased the expression of mesenchymal markers (e.g., N-cadherin, vimentin and β-catenin) and enhanced the expression of the epithelial marker E-cadherin, thus inducing a MET-like phenotype in glioma cells.

Recently, miR-211 is shown as a suppressor of melanoma invasion, and its expression is silenced or selected via suppression of the entire melastatin locus during human melanoma progression [12]. The present study is the first report showing the anti-proliferative and anti-invasive role of miR-211 in glioma cells. We found that miR-211 overexpression inhibits MMP-9 activity and downregulation of MMP-9 (pM) further promotes miR-211 expression *in vitro* and *in vivo*. Further, we observed that MMP-9 overexpression does not have much effect on the basal expression of miR-211 in glioma cells but has a significant role in the methylation-mediated silencing of the miR-211 promoter. Thus, we assume that the role of MMP-9 overexpression in methylation of the miR-211 promoter may be necessary to ensure continued silencing of miR-211 in malignant GBM cells.

Mammalian DNA is predominantly methylated at the C-5-position of complimentary CpG bp by concerted action of three DNA methyltransferases namely, Dnmt1, Dnmt3a, and Dnmt3b [59]. This epigenetic modification is essential for mammalian development and its aberrations lead to a variety of diseases including cancer [60-62]. The miRs harboring CpG islands undergo methylation-mediated silencing, a characteristic of many tumor suppressor genes. This epigenetic modification of miRNA promoters serve as unrealized potential targets of cancer therapy. Evidence presented in this study points at the possible connection between miR-211 overexpression and MMP-9 downregulation; however, future studies are required to further investigate this connection.

Elevated plasma levels of MMP-9 have been found in lung cancer, breast cancer and liver cancer during radiotherapy [63, 64]. Sublethal doses of radiation have been shown to induce MMP-9 expression in medulloblastoma, meningioma, and hepatocellular carcinoma [51, 63, 65]. Moreover, MMP inhibitors have been shown to block radiation-induced invasion and metastasis of human pancreatic cancer cells [66]. Further, a case report study of a 13-year-old boy with GBM showed local recurrence fourteen months after surgical resection followed by radiotherapy and temozolomide chemotherapy. The clinical outcome in this case was correlated with increased expression of MMP-9 [23]. Further, miRNAs are emerging as a class of endogenous gene modulators that control protein levels, thereby adding a new layer of regulation to the DNA damage response [67]. Recently, several miRNAs were shown to influence sensitivity to chemo- or radiotherapy and indeed, different miRNAs have been found to predict sensitivity to anti-cancer treatment such as miR-30c, miR-130a and miR-335 are downregulated in various chemoresistant cell lines [68] and the hsa-Let-7g and hsa-miR-181b are strongly associated with response to 5-fluorouracil-based antimetabolite S-1 [69]. Recent studies showed that the miRNA-221 and miRNA-222 may present novel targets for radiosensitization due to their regulation of the PTEN/AKT pathway in tumor cells [70]. Given the insensitivity of some GBMs to radio- and chemotherapy (temozolomide) and the hypothesis that glioma stem cells cause resistance to drug therapy, we investigated whether miR-211 endows radiosensitivity to glioma cancer stem cells by synergistic downregulation of MMP-9. In combination with miR-211 overexpression and inhibition of MMP-9, even the sublethal dose of ionizing radiation (5 Gy) was sufficient to cause significant disruption of neurospheres and DNA fragmentation in glioma stem cells. The MDR1/P-gp-mediated-Rhodamine 123 drug efflux activity was also shown to be suppressed with miR-211 overexpression, thus demonstrating its role in increased drug retention by chemosensitization of glioma stem cells.

In conclusion, our study suggests that the oncogene MMP-9 could be negatively regulated at the post-transcriptional level by miR-211. Furthermore, we show that the miR-211 induced apoptosis and had anti-proliferative and anti-invasive effects in glioma cells (Fig. 4D). These data along with our data from the GBM tissue array and intracranial xenograft model of brain tumors may support a strategy for targeting the miR-211/MMP-9 interaction or rescuing miR-211 expression as a new therapeutic application to treat GBM patients in the future.

## MATERIALS AND METHODS

### Cell lines, cancer stem cell (CSC) neurospheres, transfection, and ionizing radiation (IR)

U87 cells obtained from the National Cancer Institute (NCI) (Frederick, MD) and 4910 xenograft cell line (a kind gift from Dr. David James, University of California, San Francisco) were maintained in DMEM and RPMI-1640 respectively [71]. The primary culture of 4302 derived from a human GBM specimen (a kind gift from Dr. Jeremy Rich, Cleveland Clinic, Cleveland, OH) and parental 4910 cells were subjected to cancer stem cell (CSC) neurosphere formation as described previously [71, 72]. Primary neurospheres were obtained in five to six days and were subsequently disturbed by trituration; the single cell suspension obtained was then plated for secondary neurospheres formation. Neurospheres of around 8-12 passages were used for this study. All transfection experiments were performed with FuGene *HD* transfection reagent (Roche, Indianapolis, IN) according to the manufacturer's protocol. Briefly, plasmid/shRNA was mixed with FuGene *HD* reagent (1:3 ratio) in 200-500 μL of serum free-medium and left for 30 min at room temperature (RT). The complex was then added to a 6-well or 100-mm plate containing 1 mL or 5 mL of serum-free medium, and around 2 μg plasmid per mL of medium was used. After 8 h of transfection, complete medium containing 10% FBS was added and the neurospheres were incubated for 48-72 h. For combination treatments, a radiation dose of 5 Gy was given using the RS 2000 Biological Irradiator X-ray unit (Rad Source Technologies Inc., Boca Raton, FL) after 48 h of transfection, and the irradiated neurospheres were incubated for 5 days.

### Chemotherapy compound

Temozolomide (T2577) was purchased from Sigma (St. Louis, MO). The 4302 and 4910 CSC were treated with temozolomide (500 μM). Cytotoxicity was determined after 48 h of incubation.

### Glioblastoma (GBM) tumor specimens

Glioblastoma specimens (Grade IV) were obtained during autopsies of glioblastoma patients within 24 h of death or from patients who underwent surgery at Saint Francis Medical Center (Peoria, IL). All samples were collected under protocols approved by the UICOMP (Peoria, IL) Institutional Review Board.

### Plasmids and shRNA constructs, antibodies and reagents

We purchased PLKO-mcherry-luc-puro-miR-211 construct for the overexpression of miR-211 and empty vector (EV) from Addgene (Cambridge, MA). The shRNA construct designed to knockdown MMP-9 (pM) and the MMP-9 overexpression construct (MMP-9 OE) were generated in our laboratory [30]. Control cells were processed in the same way as treated cells and were incubated with equal volumes of FuGene *HD*. The following antibodies: anti-MMP-9, Cyclin D1, p-Cdc2 p34 (Thr14/Tyr15), p-p53 (ser 15), p53, pERK1/2, ERK, Caspase-9 p35, Apaf-1, Bax, Bcl-2, Bcl-xL, Smad-1, Smad-3, β-catenin, E-cadherin, N-cadherin, Vimentin and CD133 were purchased from Santa Cruz Biotechnology (Santa Cruz, CA). Caspase-3 and TGFβ-1 were purchased from Cell Signaling (Beverly, MA), and PARP-1 was purchased from VWR (San Dimas, CA). A protease inhibitor cocktail was purchased from Sigma (St. Louis, MO). Protein concentration was estimated using the bicinchoninic acid (BCA) assay (Pierce, Rockford, IL).

### Immunoblotting

Immunoblot analysis was carried out using equal amounts of protein resolved on SDS-PAGE, transferred onto nitrocellulose membrane (Biorad, Hercules, CA), incubated with 1:500 dilution of primary antibodies, and subsequently incubated with 1:1000 dilution of species-specific, HRP-conjugated secondary antibody as per standard protocols [65].

### Fluorescence-activated cell sorting (FACS) analysis, TUNEL assay, apoptotic DNA fragmentation analysis and mitochondrial apoptosis detection

Progression through different cell cycle phases was monitored by flow cytometric analysis of DNA content in cell populations stained with propidium iodide and was carried out with a fluorescence-activated cell sorter (FACS Caliber flow cytometer; Becton Dickinson). The percentages of treated cells in various cell cycle phases (G0/G1, S, and G2/M) were analyzed by FACS as described previously [65]. The TUNEL assay was done using an *in situ* cell death detection kit (Roche Applied Science, Indianapolis, IN) according to the manufacturer's protocol. Briefly, treated cells were fixed for 24 h in 8-well chambered slides with 4% paraformaldehyde in 0.1 M phosphate buffer (pH 7.4). Next, cells were incubated with a TUNEL reaction mixture for 60 min at 37°C in a humidified incubator. The slides were washed three times with PBS and the incorporated biotin-dUTP was detected under a fluorescence microscope. Mitochondrial membrane potential changes were assayed with MitoLight dye according to manufacturer's instructions (Millipore, Danvers, MA). 48 h after treatment, cells were incubated with pre-diluted MitoLight solution for 30 min. Cells were washed twice with 1X incubation buffer, mounted with cover slips, and observed immediately under a fluorescence microscope. The apoptotic DNA fragmentation analysis was done using TACSTM DNA Laddering Kit (R&D Systems, Minneapolis, MN) following the manufacturer's instructions. About 1×10^6^ cells were lysed, and the DNA was extracted under stable conditions. The DNA was estimated, and around 200 ng of DNA was electrophoresed on 1.5% TreviGel 500 gel in 1X TAE buffer and visualized using ethidium bromide staining.

### Glioma CSC migration and Matrigel invasion assay

The migration of CSC neurospheres and Matrigel invasion were carried out using previously established protocols [39, 52].

### Gelatin zymography

MMP activity in conditioned medium was determined by gelatinase zymography as we described previously [73]. After 48 h of treatment, the glioma cells and CSC were incubated overnight with serum-free medium. Conditioned medium containing equal amounts of protein were electrophoresed on 10% SDS-polyacrylamide gels containing 1.5 mg/mL gelatin. The gels were stained with 0.1% amido black in 10% acetic acid and 10% isopropanol and subsequently destained for 1 hr. Gelatinolytic activity was identified as clear zones of lysis against a dark blue background.

### MiR-quantitative real time-PCR (RT-PCR)

Total RNA was isolated using TRIZOL reagent (Invitrogen, Carlsbad, CA) according to the standard protocol. For miRNA analysis, 10 ng of template RNA diluted to a final concentration of 2 ng/μL was reverse transcribed using first-strand cDNA synthesis kit and miR-211-specific RT primers from the miRCURY LNATM microRNA polymerase chain reaction system (Exiqon, Vedbaek, Denmark) according to the manufacturer's instructions. The miR-211 transcript levels were examined by RT-PCR using the CFX96™ Real Time System (Bio-Rad) in triplicate and the SYBR Green PCR Master Mix (Applied Biosystems, Framingham, MA). The following PCR conditions were used: 95°C for 10 min, followed by 50 cycles at 95°C for 10 sec and 60°C for 30 sec. The expression level of miR-211 in pM and MMP-9 OE treatments was normalized to control (untreated) cells, and the fold change was calculated using 2-ΔΔCt, where ΔΔCT = ΔCT of treatment – ΔCT of control.

### Bisulfite modification of genomic DNA and methylation-specific polymerase chain reaction

Genomic DNA was isolated from 4910 and U87 control, pM, 5-Aza-CdR (5 μm, Sigma, St. Louis, MO) and MMP-9 OE-treated cells and GBM grade IV specimens using a DNeasy tissue kit (Qiagen). Bisulfite reaction was carried out on 5 μg of genomic DNA using EpiTect Bisulfite Kit (Qiagen) following the supplied protocol. The MSP for aberrant gene promoter methylation was performed as previously described [74]. MSP was carried out using bisulfite modified DNA (200 ηg) in a total volume of 25 μL. We used the following MSP primers: methylated-MSP (M) 5'-TGGTTTATTAGGAAAAGTTGTAGATTTTC-3' (sense) and 5'-CTACCCTACTCAAAACCCTAAACG-3' (antisense); unmethylated-MSP(U) 5'- TGGTTTATTAGGAAAAGTTGTAGATTTTT-3' (sense) and 5'- TACCCTACTCAAAACCCTAAACAAA-3' (antisense). PCR conditions were: 95°C for 10 min; then 40 cycles at 95°C for 45 sec, 50°C for 45 sec, and 72°C for 45 sec; and a final extension of 10 min at 72°C. Negative control MSP reactions were performed using water only as a template. PCR products were verified by 2% agarose gel electrophoresis.

### Rhodamine 123 (R123) efflux assay

R123 efflux assay was performed using Multidrug Resistance Direct Dye Efflux Assay kit (Millipore, Danvers, MA) as per manufacturer's instructions. The assay measures MDR1/P-gp-mediated efflux by the decrease in the intracellular fluorescence of rhodamine 123 (R123, a MDR1/P-pg substrate) using flow cytometry. Around 2.5×10^5^ cells were incubated for 2 h at 37°C in the R123 loading buffer. The R123 solution was removed from the extracellular medium by centrifugation, and the cells were resuspended separately into warm efflux buffer containing DMSO and 22 μM Vinblastin, a MDR1/P-pg blocker. For the negative control, the cells were incubated in ice-cold efflux buffer. After a 1 h incubation period, fluorescence of R123 was collected through a 530/30 nm bandpass on a FACS Calibur flow cytometer (Becton-Dickinson, San Jose, CA). After gating for live cells, 10,000 cells were recorded for each sample and processed by Cell Quest software (Becton-Dickinson).

### Surgical orthotopic implantation

2×10^5^ 4910 xenograft glioblastoma cells were injected intracerebrally into nude mice as previously described [75]. Tumors were allowed to grow for 10 to 12 days, and the animals were separated into groups (six animals per group). Alzet osmotic pumps (model 2004, ALZET Osmotic Pumps) were implanted for plasmid delivery (dose: 3–6 mg/kg body weight) as described previously [30]. After 6 weeks or when the control animals started showing symptoms, the animals were euthanized. The brains were collected and fixed in buffered formaldehyde. To visualize tumor cells and to examine tumor volume, the brain sections were stained with H&E.

### Immunohistochemistry

For immunohistochemical analysis, tissue sections (5–6 μm thick) were deparaffinized in xylene and rehydrated in graded ethanol solutions. Antigen retrieval was carried out with 10 mM citrate buffer (pH 6) at boiling temperature for 60 min and permeabilization in 0.1% Triton-X-100. The tissue sections were incubated with primary antibody for MMP-9, incubated with HRP-conjugated secondary antibodies, followed by DAB peroxidase substrate (Sigma, St. Louis, MO) solution, counterstained with hematoxylin, and mounted. The images were captured with an Olympus BX61 fluorescent microscope attached with a CCD camera.

### In situ hybridization (ISH)

The miRCURY LNA™ microRNA ISH Optimization Kit (FFPE) and full-length miR-211 hybridization probe were purchased from Exiqon (Woburn, MA). *In situ* hybridization was carried out as per the manufacturer's instructions [76]. In brief, paraffin-embedded tumor tissue sections were subjected to deparaffinization and the following steps were performed: proteinase-K treatment at 37°C, pre-hybridization at 55°C for 15 min, hybridization with DIG-labeled LNA miR-211 probe (50 nM) at 55°C for 60 min, stringent washes with SSC buffers at 55°C for a total of 33 min followed by DIG blocking reagent (15 min at RT), alkaline phosphatase-conjugated anti-DIG at 1:1000 dilution (60 min at RT), alkaline phosphatase-substrate; enzymatic development (120 min at RT), and nuclear fast red counterstain (5 min). The slides were mounted and air dried, and the images were captured with an Olympus BX61 fluorescent microscope attached with a CCD camera.

### Bioinformatic approach

Computational predictions of putative miR-494 binding sites in the 3^’^-untranslated region (3^’^-UTR) of MMP-9 was done using target prediction algorithms, miRanda [24] and TargetScan [25]. Human miR-211 sequences of the RFAM miRNA registry [77] were downloaded from the miRBase website (http://microrna.sanger.ac.uk/sequences/index.shtml).

### Statistics

All data are presented as means ± standard deviation (SD) of at least three independent experiments (each performed at least in triplicate). One-way analysis of variance (ANOVA) combined with the Tukey post-hoc test of means were used for multiple comparisons in cell culture experiments. Statistical differences are presented at probability levels of *p*<0.05, *p*<0.01 and *p*<0.001.
